# Hospital Readmissions for Hyperparathyroidism After Bariatric Surgery in the United States: A National Database Review

**DOI:** 10.7759/cureus.10585

**Published:** 2020-09-22

**Authors:** Fred N Qafiti, Michael A Lopez, Kandace Kichler, Joshua Parreco, Jessica L Buicko

**Affiliations:** 1 General Surgery, Florida Atlantic University Charles E. Schmidt College of Medicine, Boca Raton, USA; 2 General Surgery, University of Miami Miller School of Medicine, Lantana, USA; 3 Bariatric Surgery, University of Miami Miller School of Medicine, Lantana, USA; 4 Trauma, Florida State University, Fort Pierce, USA; 5 General Surgery, Bethesda Health Physician Group, Bethesda Hospital East, Boynton Beach, USA

**Keywords:** hyperparathyroidism, sleeve gastrectomy, readmission risk, roux-en-y gastric bypass

## Abstract

Introduction: The incidence and significance of hyperparathyroidism in patients after bariatric surgery have been established to some degree; however, the impact it has on the national healthcare system has not. We sought to assess the risk of readmission and related comorbidities in this patient population.

Methods: The Healthcare Cost and Utilization Project Nationwide Readmission Database was queried for all patients who underwent Roux-en-Y gastric bypass (RYGB) or sleeve gastrectomy (SG). Multivariate logistic regression analysis was conducted to identify factors associated with readmission for hyperparathyroidism.

Results: A total of 915,792 patients between 2010 and 2015 were queried; 43.2% had undergone SG and 56.8% had RYGB. A total of 589 patients were readmitted for hyperparathyroidism; 80.8% were female and 68% had a Charlson comorbidity index ≥ 2. Factors associated with readmission were as follows: age 45-64 years (odds ratio [OR] 1.42, p=0.001), Medicare (OR 3.01, p<0.001) or Medicaid (OR 2.61, p<0.001) insurance status, lower median household income, renal failure (OR 17.14, p<0.001), hypertension (OR 2.89, p<0.001), and deficiency anemia (OR 2.62, p<0.01).

Conclusions: Parathyroid axis monitoring may provide benefits to predictably high-risk patients. Appropriate surveillance may decrease the impact of bariatric hyperparathyroidism readmission on the U.S. healthcare system.

## Introduction

The prevalence of obesity and morbid obesity in the United States has increased dramatically in recent decades. Whereas obesity rates (body mass index [BMI]>30) have increased fivefold from 1960 to the mid-2000s, the rate of morbid obesity (BMI>40) is now estimated at 12 times of what it once was [1]. On average, 1.3 million Americans are estimated to become obese or morbidly obese annually [2].

Bariatric surgery is of paramount importance in the effective management of obesity. In 1991, the National Institute of Health Consensus Conference published indications for bariatric surgery that have remained the standard for almost three decades. The indications include motivated and well-informed patients with BMI>40, BMI>35 with associated comorbidities (diabetes mellitus, peripheral vascular disease, obstructive sleep apnea, etc.), and those for whom alternative therapies have a low probability of sustained weight loss [3].

The literature reproducibly demonstrates significant reductions in comorbidities and all-cause mortality in these patients after surgery [1]. However, bariatric intervention introduces a variety of nutritional challenges in an already metabolically compromised population. The enteric absorption of calcium decreases after bariatric interventions [4]. Parathyroid hormone (PTH) has been shown to physiologically compensate, causing additional metabolic strain on the body. Ultimately, secondary hyperparathyroidism precipitates [5,6].

Malabsorption of calcium after bypass surgery has been well documented. Few studies have explored hyperparathyroidism prevalence in exclusively restrictive bariatric surgery such as the sleeve gastrectomy (SG). No studies were found reviewing long-term rates of hyperparathyroidism readmission and treatment in bariatric patients.

## Materials and methods

The Healthcare Cost and Utilization Project Nationwide Readmission Database from 2010 to 2015 was queried for all patients who had previously underwent SG or Roux-en-Y gastric bypass (RYGB) with a readmission diagnosis of hyperparathyroidism. Historically and theoretically predictive patient variables were obtained. This includes age, sex, BMI, Charlson comorbidity index (CCI), comorbidity type, primary insurance, and median household income quartile. Additionally, non-patient variables such as hospital financial model type and hospital size were obtained for a socioeconomic comparison of health system utilization. Multivariate logistic regression was used to determine the odds ratios (ORs) for the outcomes of interest. The Nationwide Readmission Database is a publicly accessible data set that contains no direct patient identifiers. Appropriately, no Institutional Review Board approval was sought.

With this retrospective database review, we explore readmission rates for hyperparathyroidism in both RYGB and SG patients. This study broadens our understanding of the impact of shifting calcium demands in the growing bariatric population and proposes an argument for parathyroid axis monitoring.

## Results

A total of 915,792 patients had bariatric surgery of whom 43.2% had undergone SG with the remaining having had RYGB (56.8%). In total, 589 bariatric surgery patients were readmitted for hyperparathyroidism (0.1%). The majority were female (80.8%) and had a CCI≥2 (68.0%). There were 221,382 patients with a BMI≥50 (24.2%).

Multivariate regression demonstrated that patients between the ages of 45 and 64 (OR 1.4, p=0.00) and those with Medicare (OR 3.0, p<0.001) or Medicaid (OR 2.6, p<0.001) were more likely to be readmitted after bariatric surgery for hyperparathyroidism (Table 1). In addition, patients in lower median household income quartiles were more likely to be readmitted. Smaller hospital bed size was a protective factor and patients were less likely to get readmitted along with those at investor-owned hospitals. Comorbidities associated with the highest ORs for readmission with hyperparathyroidism were renal failure (OR 17.1, p<0.001), hypertension (OR 2.9, p<0.001), and deficiency anemias (OR 2.6, p<0.01).

**Table 1:** Multivariable logistic regression analysis of factors associated with hyperparathyroidism readmission after bariatric surgery * indicates p<0.05. SG, sleeve gastrectomy; RYGB, Roux-en-Y gastric bypass; CCI, Charleston comorbidity index.

Characteristics		OR	CI	p-value
Age group (years)	14-44			
	45-64	1.42	1.15-1.76	0.001*
	65+	0.86	0.63-1.18	0.363
Bariatric surgery type	RYGB			
	SG	1.02	0.86-1.22	0.792
CCI	0-1			
	≥2	1.16	0.86-1.55	0.329
Control of hospital	Not-for-profit			
	Public	0.94	0.72-1.22	0.633
	Investor-owned	0.71	0.52-0.97	0.032*
Primary payer	Private insurance			
	Medicare	3.01	2.44-3.72	<0.001*
	Medicaid	2.61	1.98-3.44	<0.001*
	Self-pay	0.29	0.08-1.06	0.062
	No charge/other	0.79	0.38-1.65	0.528
Median household income quartile	4th (wealthiest)			
	3rd	1.77	1.30-2.40	<0.001*
	2nd	2.59	1.94-3.46	<0.001*
	1st (poorest)	1.80	1.32-2.44	<0.001*
Bed size of hospital	Large			
	Medium	0.71	0.57-0.89	0.003*
	Small	0.61	0.45-0.83	0.002*

## Discussion

Parathyroid physiology in bariatrics

Calcium malabsorption and long-term impacts on bone density in bariatric patients are well characterized. The duodenum plays a large role in the absorption of calcium given its high density of membrane transporters and the naturally acidic environment required for calcium solubilization [4]. Accordingly, large decreases (34%) in calcium absorption and subsequent increases in bone resorption have been demonstrated during the first six months following RYGB surgery [7].

PTH produced by the parathyroid gland compensates for the deficiency. One study demonstrated that PTH levels are persistently higher in women who have undergone RYGB compared to matched controls both before and after six months of calcium supplementation. Notably, the measured lumbar spinal and femoral bone density in both groups were unchanged [5]. A study of 283 RYGB and duodenal switch patients demonstrated that 37% exhibited biochemical hyperparathyroidism after a median follow-up of 35 months. Of those patients, 42% demonstrated clinically symptomatic hyperparathyroidism [6]. These studies suggest overcompensation of a physiologically hyperactive parathyroid gland in malabsorptive bariatric interventions.

Sleeve gastrectomy versus RYGB

Readmission rates are frequently used as quality metrics for patient care. In this instance, we utilize them to make a high-powered assessment of the national impact of hyperparathyroidism in a large subset of bariatric patients.

Whereas calcium demands have been well studied in malabsorptive bariatric surgery, nutritional needs in restrictive operations have limited evidence. One study found no difference in hyperparathyroidism between SG and RYGB patients over a median of three years postoperatively, finding that the only determinant was menopausal age among postmenopausal women [8]. A regression study by Wei et al. of 1470 patients undergoing various types of restrictive and malabsorptive bypass procedures demonstrated that the type of surgery performed was an independent predictor of secondary hyperparathyroidism at the one-year postoperative follow-up [9].

In contrast to Wei et al.'s study, our study finds that between RYGB and GS patients, bypass surgery was not an independent predictor of readmission rates for hyperparathyroidism in our national database query. The discrepancy of findings can be reconciled by recognizing that differences in biochemical hyperparathyroidism may not necessarily dictate readmission rates for the clinical disease. Our results suggest that at the health system level, the clinical impact of these shifting endocrine demands is similar between RYGB and GS.

Socioeconomic influences

Primary insurance status and median household income quartile are reliable markers of socioeconomic status (SES). We find that they also correlate to readmission rates for hyperparathyroidism at the national level. Patients whose primary insurance is Medicaid or Medicare were less likely to be readmitted for hyperparathyroidism than those who benefit from private insurance. Patients whose median household income was within the lowest three quartiles were also less likely to present for readmission. This may be indicative of the disparity of healthcare access and usage among bariatric patients across the economic spectrum. Similar trends of nutritional deficiency have been correlated to SES. Low SES was associated with decreased preoperative ferretin levels, a marker of iron deficiency, in bariatric surgery candidates [10]. One study of 42 low-SES bariatric patients 10 years after surgery demonstrated that 47% had an iron deficiency anemia, whereas 16.6% were hypoalbuminemic [11].

Hospital factors

Hospital factors, namely, bed size and financial status, were found to correlate significantly as well. Large hospitals tend to readmit a significantly larger proportion of their bariatric patients for hyperparathyroidism. In comparison to investor-owned hospitals, public hospitals do the same. Accordingly, these hospitals may be the most effective targets for education and implementation of parathyroid axis monitoring.

Correlated comorbidities

Multiple comorbidities were found to correlate as strong independent predictors of hyperparathyroidism admission. Specifically, renal failure, hypertension, and nutritional deficiency anemia demonstrated the greatest correlation. Renal failure alone predisposes patients to secondary and tertiary hyperparathyroidism, the foundation of which is the failure of the parathyroid axis to convert vitamin D to a metabolically active form. Hypertension, although predisposing to renal failure, demonstrates a significant predictive strength that is independent of renal failure.

Deficiency anemia is a well-known sequela of the shifting nutritional needs of bariatric patients. Iron deficiency is well documented in bariatric patients [10,11]. Anemia is a surrogate of overarching nutritional deficiency patterns in the bariatric patient, which exacerbate the demand on the parathyroid axis. Understandably, these patients become high risk for hyperparathyroidism readmission.

Study limitations

The limitations of this study include the potential for discrepancy in definition of diagnoses between institutions. The Nationwide Readmission Database is based on International Classification of Diseases coding. Accordingly, the classification of variables, such as hyperparathyroidism, CCI, deficiency anemia, and hypertension, is based on clinician judgement, with an inevitable variation between institutions. Furthermore, we were unable to delineate between subtypes of hyperparathyroidism; follow-up studies ought to focus on identifying trends within these subclassifications.

## Conclusions

Efforts should be made to reduce readmission for patient groups at higher risk. Bariatric patients with renal failure, hypertension, and anemia would likely benefit from earlier follow-up with parathyroid axis monitoring (i.e., outpatient monitoring of calcium, vitamin D, and PTH levels). Patients who received the non-malabsorptive SG may also benefit given that hyperparathyroidism admission rates are similar between RYGB and SG groups. Monitoring is likely most beneficial for patients younger than 45 years of age.

Larger public hospitals may benefit most from proactive parathyroid axis monitoring. Their large volumes and higher risk for hyperparathyroidism readmission make them ideal candidates for the implementation of these programs. Further quality improvement studies ought to be done to explore this benefit and its application to subclassifications of hyperparathyroidism.
